# Development and application of a clinical psychological nursing training program for nursing interns based on the ADDIE model

**DOI:** 10.3389/fmed.2025.1720671

**Published:** 2026-01-28

**Authors:** Yanfang Dong, Min Pang, Xiaoqing Zhu, Shiyu Wu, Yiming Li, Yanjing Huang

**Affiliations:** 1 Department of Emergency Intensive Care Unit, The First Affiliated Hospital, Fujian Medical University, Fuzhou, China; 2 Department of Emergency, The First Affiliated Hospital, Fujian Medical University, Fuzhou, China; 3 Department of Intensive Care Unit, Gutian County Hospital, Ningde, Fujian, China; 4 Department of Emergency, National Regional Medical Center, Binhai Campus of the First Affiliated Hospital, Fujian Medical University, Fuzhou, China

**Keywords:** ADDIE model, Delphi method, empathy, nursing education, resilience

## Abstract

**Objective:**

To develop and apply a clinical psychological nursing training program for nursing interns based on the ADDIE model, aiming to enhance their psychological nursing competencies.

**Methods:**

From August 1, 2023, to February 1, 2025, the five phases of the ADDIE model (Analysis, Design, Development, Implementation, and Evaluation) were adopted. First, the current status of clinical training was surveyed, and the content and format of training were analyzed to draft a preliminary training program. Then, two rounds of Delphi expert consultations (involving 14 experts) were conducted to optimize and finalize the program. Subsequently, a single-group pre-post intervention study was conducted, where the program was implemented among 75 nursing interns. Its effectiveness was evaluated using the Connor-Davidson Resilience Scale (CD-RISC), the Jefferson Scale of Empathy (JSE), and a self-designed knowledge assessment test.

**Results:**

The questionnaire response rates for both rounds of expert consultations were 100%. For first-level items, the mean importance scores ranged from 4.41 to 4.91, with coefficients of variation (CV) of 4.40–15.72%. For second-level items, the scores ranged from 3.78 to 4.93, with CVs of 4.56–19.42%. The finalized training program included 6 key aspects and 7 training formats.

**Conclusion:**

The program is scientifically sound, innovative, with easily understandable content and diverse formats. It shows significant training effectiveness and has guiding value for clinical training of nursing interns.

## 1 Introduction

The bio-psycho-social medical model emphasizes a patient-centered holistic nursing approach. Psychological nursing plays a crucial role in modern nursing practice and has become a core subject in nursing education (1). A multicenter study in China showed that psychological nursing interventions can reduce patients’ post-operative anxiety scores by 30–40% and improve their treatment compliance by 25% (2), which further confirms its clinical value. Effective psychological nursing cannot only alleviate patients’ anxiety but also enhance their psychological resilience and self-management capabilities. However, nursing interns mainly acquire psychological nursing knowledge through the Nursing Psychology course. Although this course is included in the academic curriculum, its broad content and diverse application scenarios lead to limited mastery and practical application of the knowledge among nursing interns (3)—a phenomenon also reported in international studies, where only 38.6% of nursing interns can independently apply psychological nursing skills in clinical settings (4). During clinical internships, there is an obvious lack of systematic and continuous psychological nursing education, which requires focused attention and targeted interventions. As an extension of classroom learning, clinical nursing education should strengthen psychological nursing training to improve nursing interns’ competencies in this field.

The ADDIE model was developed in 1975 by the Center for Educational Technology at Florida State University for the U.S. military. It is a systematic instructional design framework (5), consisting of five phases: Analysis, Design, Development, Implementation, and Evaluation (6). This learner-centered model is practical and reliable, and is widely used in nursing education to accurately identify problems and optimize training programs (7). This study applied the ADDIE model to develop a clinical psychological nursing training program for nursing interns, aiming to establish a scientific training approach that can improve training quality and enhance nursing interns’ psychological nursing competencies. The detailed report is as follows.

## 2 Materials and methods

### 2.1 Establishment of the research team

The research team was composed of 8 members, including 1 psychology professor (responsible for program guidance and supervision), 1 chief nurse (responsible for clinical teaching management), 4 nurse supervisors (responsible for program development, implementation, interviews, and expert consultations), 1 registered nurse, and 1 nursing master’s student (responsible for data collection and analysis). All members had relevant training experience or clinical practice experience.

### 2.2 Development of the training program

**FIGURE 1:**
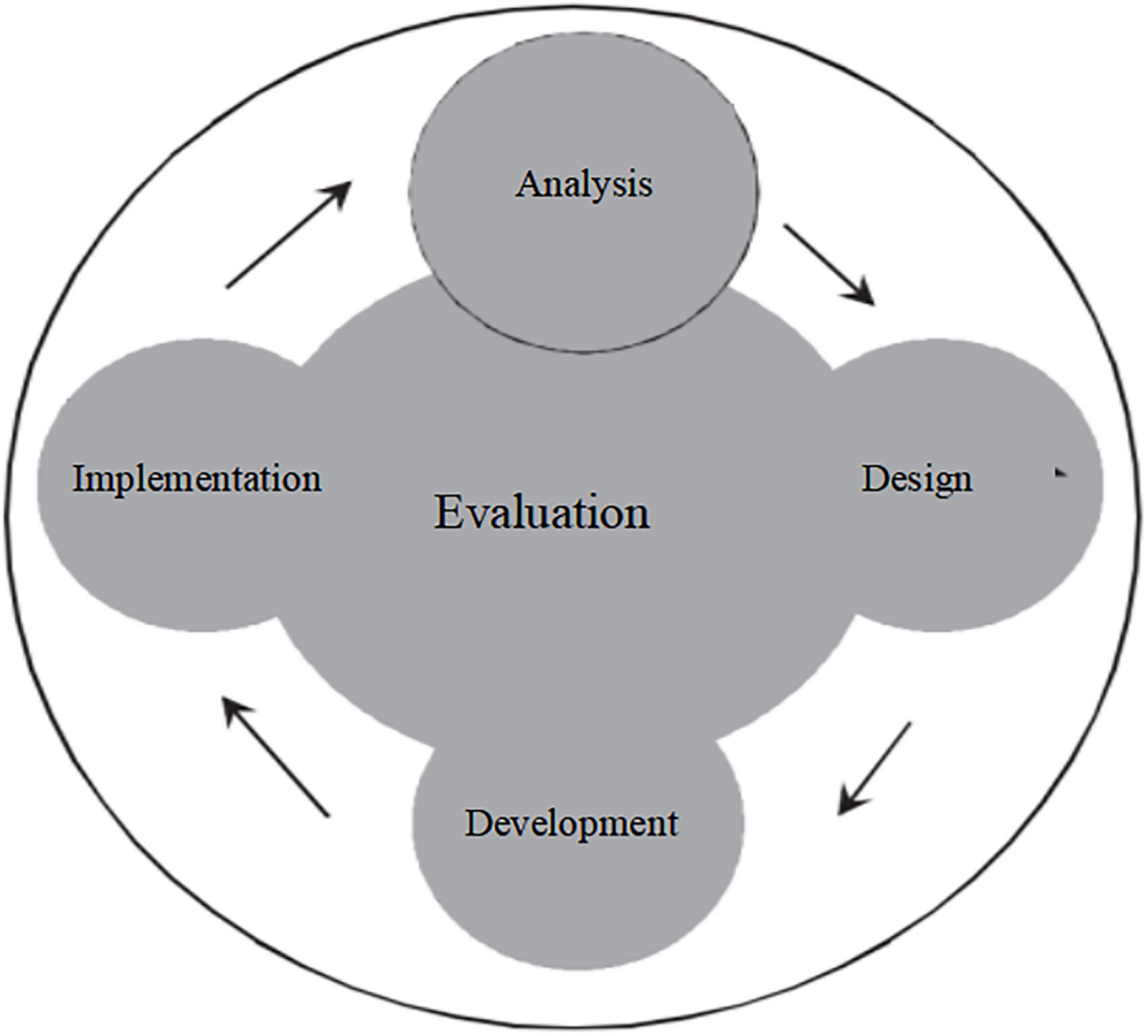
The ADDIE model.

**FIGURE 2:**
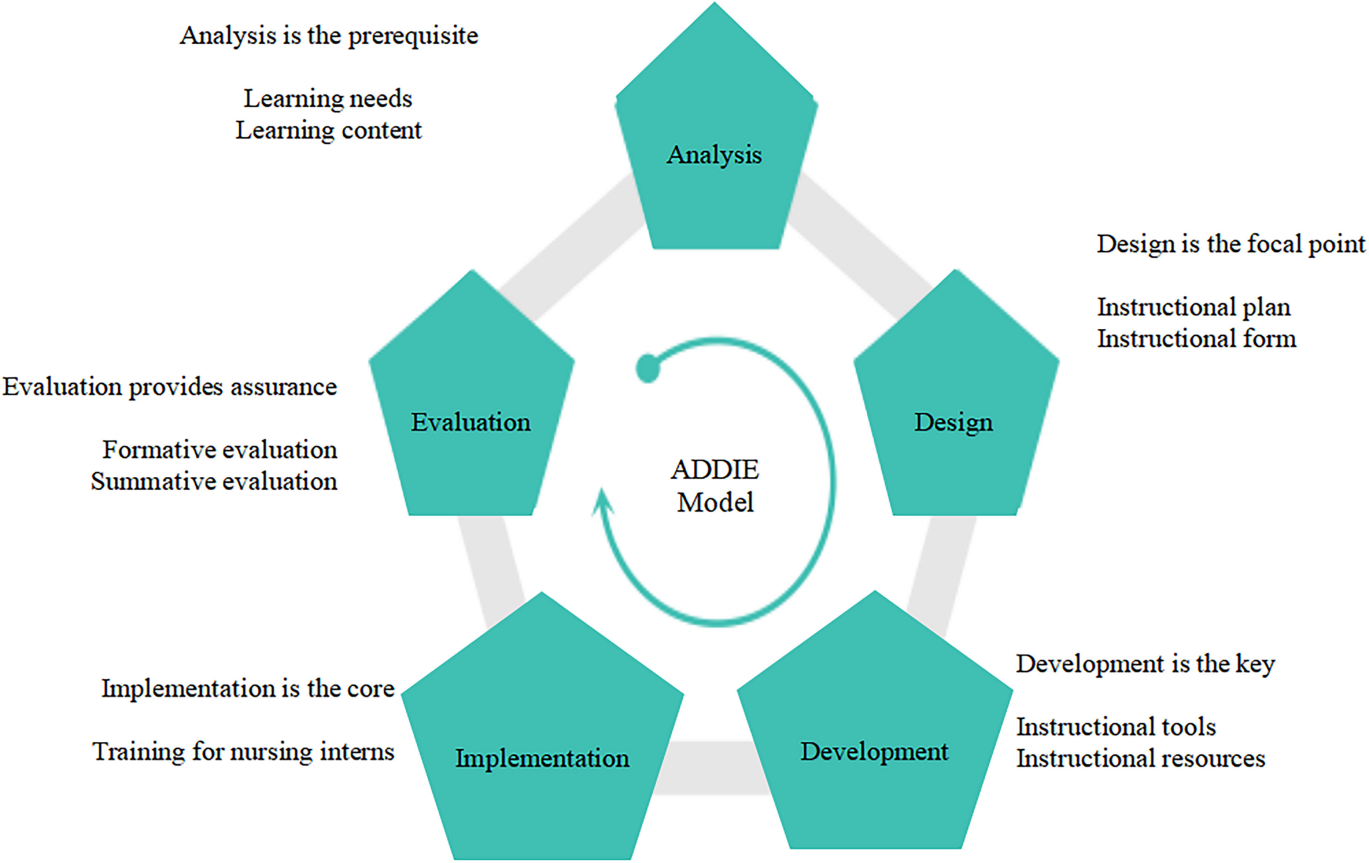
Design model for psychological nursing training among nursing interns.

The five phases of the ADDIE model are interrelated. Each preceding phase lays the foundation for the subsequent one, and the Evaluation phase runs through the entire process (Figure 1). Based on this framework, the study developed a design process for psychological nursing training of nursing interns (Figure 2).

#### 2.2.1 Analysis phase

This phase focused on assessing the training needs, current status, and objectives of clinical psychological nursing training for nursing interns. From 2023 to 2024, semi-structured interviews and questionnaires were administered to undergraduate nursing interns of the 2021 cohort who were undertaking their internships at a tertiary hospital in Fujian Province. The qualitative interview protocol was pre-developed by the research team (including 1 psychology professor and 2 senior nurse supervisors) and finalized after 2 rounds of expert review; it included 5 core modules (prior training experience, perceived importance of psychological nursing, training needs, expected content, preferred formats) with 3–4 follow-up probes per module, and each interview was audio-recorded (with informed consent) and transcribed verbatim within 24 h to ensure data integrity. Convenience sampling was used, with the following inclusion criteria: (1) having completed undergraduate theoretical coursework and currently interning at the hospital; (2) The study involving human participants (nursing interns) was reviewed and approved by the Ethics Committee of the First Affiliated Hospital of Fujian Medical University [Approval No. MACTA, ECFAH of FMU (2025)928]. All procedures performed in this study were in accordance with the ethical standards of the institutional research committee and the 1964 Helsinki Declaration and its later amendments or comparable ethical standards. Informed consent was obtained from all individual participants included in the study. Before initiating data collection (semi-structured interviews, questionnaires) and training implementation, the research team provided detailed explanations of the study purpose, procedures, potential risks, and rights (e.g., voluntary participation, right to withdraw at any time without adverse consequences, and confidentiality of personal information) to all eligible nursing interns.

Exclusion criteria: (1) having interrupted the internship; (2) having severe psychological issues or communication barriers.

The interview outline, finalized through team discussions, covered the following topics: (1) Have you participated in clinical psychological nursing training before? (2) How important is psychological nursing in clinical practice? (3) Do you think there is a need for psychological nursing training during your internship? (4) What content do you expect to be included in the training? (5) What training formats do you prefer?

#### 2.2.2 Design phase

##### 2.2.2.1 Literature review

Using keywords such as “ADDIE model,” “nursing interns,” “psychological nursing,” “training,” “program development” and their English equivalents, we searched databases including PubMed, Embase, Cochrane Library, Web of Science, and CINAHL. The content and formats of psychological nursing training programs for nursing interns reported in the literature were analyzed. Combined with the results from the Analysis phase, a preliminary training program was drafted.

##### 2.2.2.2 Expert consultation

The Delphi method (8) was adopted, and 14 experts were selected based on the following criteria: having ≥ 10 years of experience in clinical psychological nursing, nursing education, or nursing management; holding a bachelor’s degree or higher; having an intermediate or senior professional title; and being willing to actively participate in the consultation.

The expert consultation questionnaire included two parts: a Personal Information Form (collecting demographic information, judgment basis, and familiarity level of experts) and an Evaluation Scoring Form (using a Likert 5-point scale to assess the rationality, feasibility, and importance of the preliminary program). Two rounds of consultations were conducted via email/WeChat, with an interval of ≤ 1 month between the two rounds. After the first round of consultation, the feedback from experts was analyzed to revise the preliminary draft. The second round of consultation was conducted to obtain final feedback on the revised program. The selection criteria for the program content were: mean importance score > 3.50 and coefficient of variation (CV) < 25%.

#### 2.2.3 Development phase

Based on the results of the Analysis and Design phases, the content and formats of the training program were further developed. A team consisting of psychology experts and clinical nursing experts jointly designed the curriculum, discussed the syllabus and lesson plans, and prepared teaching slides and materials. The theoretical part of the training was delivered through face-to-face lectures and case analysis. The practical part was carried out using flipped classrooms, nursing rounds, and simulation exercises. In addition, mobile learning resources were released on the “Nursing Cloud School” platform to improve the training effect (see Table 1).

**TABLE 1:** Standardized training curriculum development for psychological nursing skills of nursing interns.

Component	Development content
Syllabus	1. Foundational theories + research advances + guidelines/consensus; 2. Case-based and problem-oriented approach; 3. Guidance on the development of psychological nursing programs; 4. Scenario simulations, case presentations, and critiques.
Teaching content	Theoretical knowledge: psychological characteristics and influencing factors of clinical patients (e.g., elderly, pediatric, maternal, terminal, medical, surgical, critical care patients) and caregivers; common negative emotions among nursing interns and corresponding coping strategies. Practical skills: psychological interviewing techniques, supportive psychotherapy, cognitive behavioral therapy, narrative nursing, and the development, implementation, and evaluation of psychological nursing plans.
Lesson planning	1. Collection of data (e.g., cases, videos, guidelines); 2. Collaborative drafting through group discussions; 3. Preparation and revision of slides.
Instructional form	1. Theory: face-to-face lectures, case analysis, and group discussions; 2. Practice: flipped classroom, nursing rounds, and simulation exercises.

CBL, Case-Based Learning; PBL, Problem-Based Learning; ICU, Intensive Care Unit (mentioned in “practical skills”). “Nursing Cloud School” refers to the mobile learning platform used in the study.

#### 2.2.4 Implementation phase

Convenience sampling was used to select 75 2021—cohort undergraduate nursing interns (with the same inclusion/exclusion criteria as those in section 2.2.1) to implement the training program. Training sessions were held every Monday afternoon, which was coordinated with the clinical practice and rotation schedule of the nursing interns. To ensure implementation fidelity, all instructors completed a 4-h standardized training (covering curriculum content, teaching methods, and simulation operation standards) before the program; standardized teaching materials (slides, case scripts, simulation checklists) were used uniformly; and 2 research team members (senior nurse supervisors) conducted on-site observation of 30% of training sessions to verify adherence to the protocol, with non-compliance rates < 5% addressed via real-time feedback. The implementation process included pre-class theoretical preparation, face-to-face lectures, Case-Based Learning (CBL), Problem-Based Learning (PBL), simulation exercises, peer teaching, expert panel discussions, post-training evaluations, and attendance recording.

#### 2.2.5 Evaluation phase

The evaluation of the training program included formative evaluation and summative evaluation. The formative evaluation focused on assessing the practicality of the course content, the effectiveness of teaching methods, and the participation level of nursing interns. The summative evaluation aimed to assess the content design, training formats, instructional techniques, interaction between teachers and students, training scheduling, learning support, and difficulty level of the program. The formative evaluation was conducted using post-class questionnaires, while the summative evaluation was carried out through post-training questionnaires.

Assessor blinding was not implemented in this study, as the training adopted a single-group pre-post design (all participants received the same intervention), and assessors needed to confirm participants’ pre-class preparation and post-training skill performance (e.g., simulation exercise operation) to ensure evaluation accuracy. To minimize potential bias, all assessors used standardized scoring criteria (e.g., psychological nursing knowledge test answer keys, simulation skill checklists) and 2 independent assessors reviewed 20% of the evaluation data, with an inter-rater reliability of ICC = 0.91, confirming consistency.

##### 2.2.5.1 Detailed description of evaluation tools

To assess the improvement of nursing interns’ psychological nursing competencies, three tools were used, with detailed parameters as follows:

###### 2.2.5.1.1 Connor-Davidson Resilience Scale

Number of items: 25 items (e.g., “I can adapt to unexpected changes,” “I can recover quickly from setbacks”) (9).

Answer choices: 5-point Likert scale, with scores ranging from 0 (“Not true at all”) to 4 (“True nearly all the time”); total score range: 0–100, with higher scores indicating stronger psychological resilience.

Categories (dimensions): 5 dimensions, including tenacity (8 items, e.g., “I can persist in the face of difficulties”), strength (9 items, e.g., “I have confidence in my ability to cope with problems”), optimism (4 items, e.g., “I believe things will get better”), adaptability (2 items, e.g., “I can adjust my strategies when facing obstacles”), and support-seeking (2 items, e.g., “I am willing to ask for help when needed”).

Reliability/validity: Cronbach’s α = 0.89, test-retest reliability = 0.87 [7], which has been validated in Chinese nursing populations [20].

###### 2.2.5.1.2 Jefferson scale of empathy-health professionals

Number of items: 20 items (e.g., “I try to understand what patients are going through emotionally,” “I pay attention to patients’ non-verbal cues”) (10).

Answer choices: 7-point Likert scale, with scores ranging from 1 (“Strongly disagree”) to 7 (“Strongly agree”); total score range: 20–140, with higher scores indicating stronger empathy competence.

Categories (dimensions): 3 dimensions, including perspective-taking (8 items, e.g., “I can put myself in patients’ shoes”), emotional care (7 items, e.g., “I care about patients’ emotional needs”), and role reversal (5 items, e.g., “I consider how I would feel if I were the patient”).

Reliability/validity: Cronbach’s α = 0.75 [8], which is widely used in evaluating empathy of nursing and medical professionals worldwide (11).

###### 2.2.5.1.3 Self-designed psychological nursing knowledge assessment test

Number of items: 30 items, all single-choice questions (4 options per item, only 1 correct answer) (12).

Answer choices: Each correct answer scores 3–4 points (adjusted by item difficulty), with a total score of 100; higher scores indicate better mastery of psychological nursing knowledge.

Categories (dimensions): 3 dimensions, including psychological foundations (10 items, e.g., “Which theory is the basis of supportive psychotherapy?”), patients’ psychological characteristics (10 items, e.g., “What are the main psychological characteristics of critically ill patients in ICU?”), and psychological intervention techniques (10 items, e.g., “Which method is suitable for alleviating terminal patients’ anxiety?”).

Reliability/validity: Content validity was verified by 7 experts (3 clinical psychologists, 4 senior emergency nurses with > 10 years of experience), with item-level content validity index (I-CVI) > 0.86 and average scale-level content validity index (S-CVI/Ave) = 0.96 [9]; Cronbach’s α = 0.82 in this study.

### 2.3 Statistical methods

SPSS 26.0 software was used for data analysis. Categorical variables were expressed as *n* (%), and continuous variables were reported as mean ± standard deviation. The sample size of 75 nursing interns was determined based on *a priori* power analysis (α = 0.05, power = 80%, Cohen’s *d* = 0.5 for psychological nursing competency outcomes) and consistency with similar single-group pre-post intervention studies (13), with 75 exceeding the minimum required sample size of 64 to ensure sufficient statistical power. Before conducting paired *t*-tests, the Shapiro-Wilk test verified the normality of continuous data, confirming compliance with the normal distribution and satisfying the statistical assumptions of the paired *t*-test. The response rate of experts was used to measure their engagement. The authority of experts was calculated using the Cr coefficient, which was based on the judgment criteria and familiarity levels of experts (see Tables 2, 3). The statistical significance level was set at *p* < 0.05. Additionally, to quantify the practical significance of the training intervention, Cohen’s *d* was used as the effect size indicator. For paired pre-post data, the pooled standard deviation method was applied, with the formula: *d* = (M_post − M_pre)/√[(SD_pre^2^ + SD_post^2^)/2; where Mpre and Mpost represent the pre-training and post-training mean scores, respectively, and SDpre and SDpost represent the corresponding standard deviations. The criteria for interpreting effect size magnitude followed Cohen (1988): *d* < 0.5 (small effect), 0.5 ≤ *d* < 0.8 (medium effect), and *d* ≥ 0.8 (large effect). Cohen’s d was calculated to quantify the practical significance of the training effect (not just statistical significance). For paired pre-post data, Cohen’s *d* > 0.8 was defined as a “large effect” (14), which indicates the intervention has meaningful clinical relevance for nursing interns’ competency development.

**TABLE 2:** Expert judgment criteria and weighting.

Judgment criteria	High	Medium	Low
Practical experience	0.5	0.4	0.3
Theoretical analysis	0.3	0.2	0.1
Literature reference	0.1	0.1	0.05
Intuitive perception	0.1	0.1	0.05
Total	1.0	0.8	0.5

Cr: authority coefficient (used to measure expert authority in section 3.2.2 “Expert authority and reliability”). The “Total” column represents the sum of weights for each judgment criterion.

**TABLE 3:** Expert familiarity levels and weighting.

Familiarity level	Very familiar	Moderately familiar	Generally familiar	Slightly familiar	Unfamiliar
Self-assessment	0.9	0.7	0.5	0.3	0.1

The “Self-Assessment” column refers to experts’ self-rated familiarity with the “clinical psychological nursing training program” (the core topic of this study).

## 3 Research results

### 3.1 Analysis phase

A total of 75 nursing interns (11 males and 64 females) were interviewed. None of them had participated in standardized clinical psychological nursing training before, but all of them recognized the importance of psychological nursing in clinical practice. Their expectations for the training content included theoretical knowledge, practical skills, and the latest research advances in psychological nursing. The preferred training formats were face-to-face lectures, simulation exercises, and ward rounds, and they preferred to be taught by experienced instructors.

### 3.2 Design phase

#### 3.2.1 Expert demographics

The 14 experts were from 6 tertiary care hospitals in Fujian Province. All of them held senior professional titles, including 2 full professors (14.29%), 8 nursing directors/specialists (57.14%), and 6 department head nurses (42.86%).

#### 3.2.2 Expert authority and reliability

The response rates of both rounds of expert consultations were 100%. In the first round, 18 suggestions were put forward by experts, and in the second round, only 1 suggestion was made. The experts’ judgment coefficient was 0.92, their familiarity level with the research topic was 0.88, and the overall authority coefficient (Cr) was 0.90. For first-level items of the training program, the mean importance scores ranged from 4.41 to 4.91, with CVs of 4.40–15.72%. For second-level items, the scores ranged from 3.78 to 4.93, with CVs of 4.56–19.42%.

#### 3.2.3 Expert consultation outcomes

##### 3.2.3.1 Clinical psychological nursing training content

The preliminary draft of the clinical psychological nursing training content included 6 first-level categories, 15 second-level subcategories, and 45 third-level items. After two rounds of Delphi consultations, the finalized content included 6 first-level categories, 16 second-level subcategories, and 48 third-level items. The first-level categories were foundational psychological knowledge, identification of psychological characteristics in common patient types, prevalent psychological issues among nurses, core competencies in psychological nursing, essential psychological intervention techniques, and integrated knowledge application. In the first round of expert consultation, 1 second-level subcategory (“Psychological characteristics and influencing factors of surgical patients”) and 3 third-level items were added. Experts also recommended introducing Problem-Based Learning (PBL) and Case-Based Learning (CBL) methodologies into the training. In the second round of expert consultation, the training format “scenario simulation” was revised to “flipped classroom.”

##### 3.2.3.2 Clinical psychological nursing training methods

After two rounds of expert consultation, in addition to traditional didactic teaching methods, PBL and CBL instructional models were incorporated into the training program. The PBL approach emphasizes problem orientation and active participation of nursing interns, aiming to cultivate their self-directed learning abilities and innovative exploration spirit. This approach encourages nursing interns to improve their learning outcomes through autonomous study, using formats such as small-group discussions and case analyses. The CBL approach uses clinical case simulations to replicate real-world clinical scenarios, thereby improving nursing interns’ abilities to explore and solve problems, with a particular focus on developing their clinical reasoning capabilities. The implementation methods of CBL include simulation exercises, nursing ward rounds, and role-playing activities, which collectively help nursing interns develop the abilities to identify, analyze, and solve clinical psychological nursing problems. In terms of instructional media, in addition to conventional PowerPoint presentations used in classroom teaching, experts recommended leveraging the convenience of mobile technology and incorporating platforms such as DingTalk to optimize the training effect.

### 3.3 Development phase

The finalized training program is presented in Table 4, which includes teaching slides, instructional videos, and clinical cases.

**TABLE 4:** Clinical psychological nursing training program for nursing interns based on the ADDIE model.

Category	Training format	Duration	Learning objective	Faculty qualifications	Learning method
I-1 Foundational psychological knowledge	–	–	Understand psychological theoretical frameworks; Master psychological concepts	Professors with psychology credentials and extensive research experience	–
II-1 Psychological foundations	Face-to-face group lectures	45–60 min			Lecture method (theoretical delivery)
II-2 Psychological nursing principles	Face-to-face group lectures	45–60 min			Lecture method (theoretical delivery)
I-2 Psychological characteristics of common patient types	–	–	Understand the moral traits, psychological characteristics, and their influencing factors of different patient types; master ICU patient characteristics	Clinical instructor with substantial ICU psychological intervention experience	–
II-3 Psychological characteristics and influencing factors of common patient types	Face-to-face group lectures	45–60 min			Lecture method (supplemented with cases)
II-4 Psychological characteristics and influencing factors of family members/caregivers	Face-to-face group lectures	45–60 min			Lecture method (supplemented with cases)
I-3 Common psychological issues among nursing interns	–	–	Identify prevalent psychological challenges among nursing interns; master coping strategies	Senior clinical instructors (> 10 years’ experience) with psychological intervention expertise	–
II-5 Overview of nurses’ mental health	Face-to-face group lectures	45–60 min			Lecture method (theory + current situation analysis)
II-6 Common psychological issues among nurses and coping strategies	Face-to-face group lectures	45–60 min			Lecture method (strategy deconstruction)
II-7 Common psychological issues among nursing interns and coping strategies	Small-group discussions	30–45 min			Small-group discussion method (experience sharing)
I-4 Core competencies in psychological nursing	–	–	Understand the core competencies in psychological nursing; master critical thinking	Senior clinical instructors (> 10 years’ experience) with psychological intervention expertise	–
II-8 Empathy skills	Case presentations and analysis	30–45 min			Case analysis method (competence deconstruction)
II-9 Communication skills	Nursing ward rounds	60–90 min			Clinical ward round method (practical drills)
II-10 Critical thinking	Case presentations and analysis	30–45 min			Case analysis method (thinking training)
I-5 Common psychological intervention techniques	–	–	Understand intervention methodologies; master ICU psychological crisis management	Clinical psychotherapists with extensive intervention experience	–
II-11 Overview of psychological intervention	Face-to-face group lectures	45–60 min			Lecture method (technique principle delivery)
II-12 Supportive psychotherapy	Flipped classroom	60–90 min			Flipped classroom method (learn first, practice later)
II-13 Cognitive behavioral interventions	Flipped classroom	60–90 min			
II-14 Narrative nursing	Flipped classroom	60–90 min			
II-15 Motivational interviewing	Flipped classroom	60–90 min			
I-6 Comprehensive application of knowledge	–	–	Apply psychological interventions clinically	Instructors with comprehensive psychological knowledge and teaching experience	–
II-16 Common application of psychological interventions	Simulation exercises	>45 min			Simulation teaching method (scenario-based practical operation)

ICU, Intensive Care Unit. “Professors with psychology credentials” refer to faculty with a master’s degree or above in psychology and ≥ 5 years of teaching experience. “Flipped classroom” format is explained in section 2.2.3 “Development phase.”

### 3.4 Implementation phase

The training program was implemented among 75 nursing interns (11 males and 64 females). To fit in with the clinical workflow of the department and the rotation schedule of nursing interns, Monday afternoons were set as the time for psychological nursing training. Nursing interns received course schedules in advance to facilitate their pre-class self-study. The instructional delivery combined multiple methodologies, such as didactic face-to-face lectures, CBL, PBL, and simulation exercises. Supplementary activities included peer-assisted learning and expert panel discussions, which were used to identify and address the challenges encountered during the implementation of the training program in clinical practice. After the training, a dual-assessment approach was adopted for nursing interns, which emphasized their interactive engagement during the training sessions and also included the recording of pre-class attendance and post-training evaluations.

### 3.5 Evaluation phase

#### 3.5.1 Formative and summative evaluations

The formative evaluation focused on collecting iterative feedback during the development and implementation of the training program to ensure the quality and effectiveness of instruction. The summative evaluation provided a comprehensive assessment of the overall outcomes of the program, determining whether the predetermined training objectives were achieved and providing a basis for future curriculum revisions. Detailed evaluation indicators and results are presented in Tables 5.

**TABLE 5:** Evaluation of the psychological nursing training program by nursing interns and instructors.

Evaluation dimension	Evaluation subject	Indicator	Percentage (%)
1. Satisfaction evaluation	Nursing interns (*n* = 75)	Overall satisfaction	94.56
		Course themes	93.51
		Course content	97.36
		Instructional formats	92.66
		Classroom interactions	96.88
		Instructors’ verbal delivery	92.37
	Instructors (*n* = 8)	Overall satisfaction	93.72
		Course themes	96.72
		Course content	93.19
		Instructional formats	95.42
		Classroom interactions	93.16
		Student engagement	90.12
2. Difficulty level evaluation	Nursing interns (*n* = 75)	Overall difficulty	93.21
	Instructors (*n* = 8)	Overall difficulty	95.63
3. Knowledge/skill enhancement^a^	Nursing interns (*n* = 75)	Knowledge mastery	94.64
		Doctor-patient communication skills	94.11
		Empathy skills	95.38
		Psychological nursing abilities	92.29
		Psychological resilience	96.76

^a^This dimension is only applicable to nursing interns (instructors do not participate in knowledge/skill assessment). *n*, Number of participants (nursing interns *n* = 75, instructors *n* = 8, consistent with the research team composition in section 2.1 “Establishment of the research team”).

#### 3.5.2 Evaluation of psychological nursing competency improvement in nursing interns

The survey results showed significant differences in nursing interns’ scores on the self-designed psychological nursing knowledge assessment test, the empathy scale (JSE), and the psychological resilience scale (CD-RISC) before and after the training program (*p* < 0.001). These results indicated that the training program significantly enhanced the nursing interns’ abilities to cope with stress and adversity, and their psychological resilience was notably improved after the training. In addition, the nursing interns made significant progress in all three dimensions of empathy competencies: perspective-taking, emotional care, and role reversal. Their mastery of psychological nursing knowledge also increased significantly. Detailed results are presented in Table 6.

**TABLE 6:** Comparison of nursing interns’ empathy skills, psychological resilience, and psychological nursing knowledge before and after training.

Paired comparison		*N*	Min	Max	Mean score (X ± S)	*t*-value	*P*-value	Cohen’s *d* (Effect Size)	Effect size magnitude
Self-designed knowledge assessment test	Post-training	75	55	100	92.31 ± 6.97 (95% CI: 90.37–93.89)	−19.367	<0.001	3.21	Large
	Pre-training	75	27	85	57.89 ± 13.64 (95% CI: 54.80–60.98)				
Empathy scale (JSE) scores	Post-training	75	80	130	116.54 ± 11.03 (95% CI: 114.04–119.04)	−17.221	<0.001	2.86	Large
	Pre-training	75	45	103	89.76 ± 12.03 (95% CI: 87.04–92.48)				
Psychological resilience scores (CD-RISC)	Post-training	75	75	93	83.12 ± 4.57 (95% CI: 82.59–83.65)	−15.727	<0.001	2.61	Large
	Pre-training	75	20	90	49.72 ± 13.89 (95% CI: 46.58–52.86)				

CD-RISC, Connor-Davidson Resilience Scale; JSE, Jefferson Scale of Empathy (JSE-HP: Health Professionals version); CI, Confidence Interval. *n* = 75 (all nursing interns in the implementation phase, consistent with section 2.2.4 “Implementation phase”); “pre-training” and “post-training” refer to assessments conducted 1 week before and 1 week after the 8-week training program. Cohen’s *d* = (M_post − M_pre)/√[(SD_pre^2^ + SD_post^2^)/2]; effect size magnitude: *d* < 0.5 (small), 0.5 ≤ *d* < 0.8 (medium), *d* ≥ 0.8 (Large).

## 4 Discussion

### 4.1 Necessity of the developed clinical psychological nursing training program for nursing interns

In China, research on psychological nursing mainly focuses on the mental health characteristics of patients, influencing factors, and intervention strategies, aiming to enhance the psychological nursing competencies of clinical healthcare professionals (2). As the future backbone of the nursing workforce, nursing interns often show limited interest in mental health work. This is largely because they have insufficient awareness of the risks of psychological diseases and the clinical teaching content lacks psychological nursing-related content (4). Current clinical nursing education has many shortcomings. For example, during internships, nursing interns receive inadequate training on identifying and addressing psychological issues of patients, which makes them unable to meet the psychological needs of patients. The systematic intervention fills this gap and is of great value in improving the current deficiencies in nursing interns’ psychological nursing competencies.

### 4.2 Scientific rigor, reliability, and innovativeness of the developed training program

The five phases of the ADDIE model are both independent and interrelated. The Analysis and Design phases lay the foundation for the training program, the Development and Implementation phases form the core of the program, and the Evaluation phase ensures the quality of the program. Formative evaluations can be conducted at any phase to keep the program relevant to the actual needs (7). This study integrated literature review, needs analysis, and Delphi expert consultations to ensure the feasibility and scientific rigor of the developed program. The Delphi method is a well-recognized reliable approach in nursing program development, as it facilitates consensus-building among experts and enhances the scientific quality of the training program (15). In this study, the response rates of both rounds of expert consultations were 100%, the authority coefficient of experts was > 0.7, the coefficient of variation was < 25%, and the coordination degree was high (*P* < 0.05), which indicated that the results of the expert consultations were highly reliable (16). Although the ADDIE model is widely used in nursing training (for example, it is used in blended learning to improve nursing skills), applying it to the clinical psychological nursing training of nursing interns and combining it with teaching methodologies such as PBL and CBL is a notable innovation (17). Detailed information on the Delphi consultation process (including content adjustments and format optimization) is summarized in Table 7.

**TABLE 7:** Summary of Delphi consultation process.

Modification dimension	First round status	Second round adjustment	Basis for adjustment
Training content (secondary categories)	15 secondary subcategories under 6 primary categories	16 secondary subcategories (added “psychological characteristics and influencing factors of surgical patients”)	Expert suggestion (supplement surgical patient—related content)
Training format	Included “scenario simulation”; lacked PBL/CBL integration	Revised “scenario simulation” to “flipped classroom”; added PBL/CBL methodologies	Expert suggestion (optimize interactive teaching)
Instructional media	Relied on traditional PowerPoint slides only	Added mobile learning platforms (DingTalk + “nursing cloud school”)	Expert suggestion (leverage mobile technology)
Training content (tertiary items)	45 tertiary items	48 tertiary items (added 3 items related to surgical patient psychological intervention)	Expert suggestion (improve content comprehensiveness)
Expert feedback quantity	18 valid suggestions (focused on content supplement and format optimization)	1 valid suggestion (minor adjustment to flipped classroom duration)	Consensus reached on core content/format

PBL, Problem-Based Learning; CBL, Case-Based Learning.

### 4.3 Diversified training formats enhance learning engagement and effectiveness

This training program is based on Edgar Dale’s Learning Pyramid Theory (1946), which holds that active learning methodologies (such as peer teaching) can significantly improve the knowledge retention rate of learners (18). The program integrates multiple instructional approaches, including traditional lectures, Problem-Based Learning (PBL), Case-Based Learning (CBL), simulation exercises, nursing rounds, case presentations, flipped classrooms, and small-group discussions. These diversified training formats effectively stimulate the learning motivation of nursing interns and improve their learning engagement. In nursing education, active learning methods such as PBL and CBL can significantly enhance the clinical reasoning and problem-solving competencies of nursing interns (19), and at the same time, strengthen their memory and retention of the training content, thus improving the overall training efficacy (20).

### 4.4 Demonstrated improvements in psychological resilience, empathy, and knowledge acquisition

There were significant differences (*P* < 0.001) in the scores of nursing interns on the Connor-Davidson Resilience Scale (CD-RISC), the Jefferson Scale of Empathy (JSE), and the self-designed knowledge assessment test before and after the training. This indicated that the training program effectively improved the psychological resilience, empathy skills, and psychological nursing knowledge acquisition of nursing interns. Specifically, the CD-RISC scores of nursing interns increased from 49.72 ± 13.89 to 83.12 ± 4.57, which reflected the enhancement of their psychological resilience. The JSE scores increased from 89.76 ± 12.03 to 116.54 ± 11.03, which showed that they made progress in the three dimensions of perspective-taking, emotional care, and role reversal. The scores of the self-designed knowledge assessment test rose from 57.89 ± 13.64 to 92.31 ± 6.97, which confirmed that they had acquired a large amount of psychological nursing knowledge. Notably, all outcome measures showed large effect sizes (Cohen’s *d* = 2.61–3.21), exceeding the threshold for “large effect” (*d* > 0.8). This confirms the training program’s strong practical impact—for example, the CD-RISC (resilience) had *d* = 2.61, meaning the training improved resilience by a magnitude that is clinically meaningful for interns coping with emergency department stress. These empirical findings verify the effectiveness of the developed program, which is consistent with the existing evidence that targeted training can enhance the psychological resilience and empathic competencies of nursing interns (11, 21). With a focus on systematic knowledge delivery and skill development, the training program helps nursing interns develop the ability to independently formulate psychological care plans through scenario simulation, and at the same time, strengthens their clinical preparedness to understand and address the mental health needs of patients (13).

### 4.5 Limitations and future research directions

Although this study has achieved significant results, it still has three limitations that need to be considered. First, only 14 experts participated in the Delphi consultations, with a limited number of clinical psychology specialists among them. This may affect the comprehensiveness of the training content. Second, this study was conducted in a single hospital, and the sample size of nursing interns was only 75. This may lead to selection bias and limit the generalizability of the research results. In addition, the study only used self-reported measures to evaluate the training effect, and there was no longitudinal follow-up. Therefore, it is impossible to evaluate the durability of the training effect. In future studies, multicenter trials with larger sample sizes should be conducted, and more psychologists should be invited to participate in the optimization of the training program. Longitudinal studies should also be carried out to track the clinical performance of nursing interns 6–12 months after the training. Moreover, researchers should explore the integration of digital tools (such as mobile applications) into psychological nursing training to further improve the practicality and sustainability of the training program.

## 5 Conclusion

This ADDIE-based developed clinical psychological nursing training program effectively enhances nursing interns’ psychosocial care competence, as evidenced by significant improvements in resilience, empathy, and knowledge. Its systematic development and diverse formats (simulation, mobile learning) address key gaps in current training, making it a valuable model for clinical nursing education in China. Future multi-center studies with objective evaluations will further validate its scalability and long-term effectiveness.

## Data Availability

The original contributions presented in this study are included in the article/supplementary material, further inquiries can be directed to the corresponding author.
